# Knowledge, Attitude, and Practices Towards Gut Microbiota Among Undergraduate Students of Health Sciences in a Tertiary Care Center, Tamil Nadu

**DOI:** 10.7759/cureus.95607

**Published:** 2025-10-28

**Authors:** Yasmin M, Sangeetha Munuswamy, Revathi Murugesan, Shreeram Astic Deshpande

**Affiliations:** 1 Microbiology, Karpagam Faculty of Medical Sciences and Research, Coimbatore, IND; 2 Anatomy, Karpagam Faculty of Medical Sciences and Research, Coimbatore, IND

**Keywords:** attitude, gut health, knowledge, microbiota awareness, non communicable diseases, practices

## Abstract

Introduction

Gut health roughly entails all factors that sustain the normal physiology of the gastrointestinal system. Factors like food habits, extensive antibiotic misuse have profound effects on the compositional balance of gut microbiota, thereby compromising its beneficial effects on gut health. The interplay of these factors has certainly placed the younger generation at risk of developing non-communicable diseases.

Objectives

To assess the knowledge of undergraduate health science students by the Microbiota Awareness Scale, and attitude and practices of health science students regarding gut microbiota and their significance with course of study.

Methodology

Our study was a cross-sectional descriptive study. Knowledge, attitude and practices towards gut microbiota were collected by a questionnaire using Google Forms from all participants. The questionnaire contained demographic details like age, gender, course and year of study and questions regarding knowledge, attitude and practices towards gut health. Knowledge was assessed using a prevalidated Microbiota Awareness Scale; attitude and practices using a validated self-structured questionnaire. Data were entered in an Excel sheet (Microsoft, Redmond, WA, USA) and results were analyzed using SPSS software (IBM Corp., Armonk, NY, USA).

Results

Our study had 902 participants who responded to the questionnaire which included 410 undergraduate medical students, 155 allied health science students of Karpagam Faculty of Medical Sciences and Research, Coimbatore, Tamil Nadu and 337 nursing students of Karpagam Nursing College, Coimbatore, Tamil Nadu, India. Around half of the participants (n=463, 51.33%) had adequate knowledge and the remaining (n=439, 48.66%) participants had inadequate knowledge. Nearly half of the participants (n=449, 49.77%) had a positive attitude, 234 (25.94%) participants had a neutral attitude and the remaining 219 (24.27%) participants had a negative attitude towards probiotics and prebiotics. Four hundred ninety-seven (55.09%) participants engage in practices that support a good gut microbiota, 267 (29.60%) participants engage in habits that neither benefit nor harm their gut microbiota and 138 (15.29%) participants engage in habits that negatively impact their gut microbiota.

Conclusion

Half of the surveyed population had adequate understanding of gut microbiota and the remaining participants had inadequate knowledge on gut microbiota. Almost the same proportion of participants who had adequate knowledge also have positive attitudes and hence exhibit behaviors that support good gut health. This study emphasizes that bridging the knowledge gap would promote better attitudes and practices towards factors that influence gut microbiota and instigate a change in behavioral patterns towards gut health preservation.

## Introduction

The term “microbiome” refers to the group of symbiotic and pathogenic microorganisms that are present in the body [1]. The term “microbiota” denotes to all microorganisms (bacteria, fungi, viruses, protozoa and archaea) that colonize specific areas in the host organism [2,3]. In the human body, typically consisting of around 30 trillion human cells, there are an estimated 38 trillion microbial cells [4]. The composition of the microflora in the human body is unique to each individual and is bound to be influenced by factors such as mode of childbirth, antibiotic use and socioeconomic status, in addition to diet and environment, throughout one’s lifetime [5,6].

Lifestyle diseases are on an increasing trend globally. Scientific explorations of parameters of gut health are expanding in recent times which shows the importance of maintaining good gut health. Gut health roughly entails all factors that sustain the normal physiology of the gastrointestinal system. The gut-brain axis is an established relationship backed by decades of research. Recent advancements have correlated gut microbiota and higher mental functions like memory, learning, stress, emotions, neurological disorders, allergic responses, inflammatory bowel disease and development of non-communicable diseases like obesity, cardiac diseases, liver diseases and cancer [7-12].

Factors like food habits and extensive antibiotic misuse have profound effects on the compositional balance of gut microbiota, thereby compromising its beneficial effects on gut health. The interplay of these factors has certainly placed the younger generation at risk of developing non-communicable diseases (NCDs) with potentially life-threatening complications, especially in the developing parts of the world [13].

According to the WHO, 18 million people died from NCDs in 2021 before 70 years of age; low- and middle-income countries contribute to 82% of the statistics. There were an estimated 10 million deaths due to cancer and 19 million deaths due to cardiovascular disease in the year 2021 alone [14]. A study by Noce et al. had shown the relationship of gut health in non-communicable diseases, with this interlink being the gut microbiota [15]. 

This study aimed to assess the knowledge, attitude and practices of undergraduate health science students regarding gut microbiota and their significance with course of study. 

## Materials and methods

Methodology

Our study was conducted among undergraduate medical and allied health science students of Karpagam Faculty of Medical Sciences and Research, Coimbatore, Tamil Nadu and nursing students of Karpagam Nursing College, Coimbatore, Tamil Nadu.

Study design and duration

Our study was a cross-sectional descriptive study, carried out for a period of one year (October 2024 to September 2025).

Inclusion and exclusion criteria

All students of undergraduate medical, allied health science and nursing students (first year to interns) were included in the study to whom the questionnaires were shared. Students who were unable to respond to the questionnaire within the study period were excluded. 

After getting approval from the Institutional Human Ethics Committee, Karpagam Faculty of Medical Sciences and Research (approval IHEC/381/Microbiology/08/2024) and permission to use the Microbiota Awareness Scale (MAS) from Özgür Önal who created the scale [16], the study was carried out after collecting informed consent from students above 18 years of age, and from parents for students below 18 years of age. Assent was obtained from the students below 18 years of age.

The questionnaire was used to assess the knowledge, attitude and practices. The questionnaire was shared using Google Forms to all the participants. It included basic demographic details of students (age, gender, course of study, year of study); a reliable prevalidated MAS that contains the questions related to gut microbiota, probiotics, prebiotics, gut-related diseases and some NCDs were used to assess the knowledge of students on gut microbiota awareness [16] in which options for question Number 17 was changed as per regional food habits after consulting the subject experts. A validated self-structured questionnaire for attitude and practice regarding gut microbiota was used as shown in the Appendix. Subject experts validated the content of the prepared questionnaire and it was pilot tested with 20 participants similar to our study population, after which we administered the questionnaire to collect data on attitude and practice.

The mean knowledge score of all participants was determined; participants' scores equal to or above the mean score were considered adequate, and scores less than the mean score were considered inadequate. Mean score was determined for attitude and practice. Scores more than the mean for attitude were considered as positive, scores equal to the mean and less than the mean were considered as neutral and negative attitudes respectively. Scores more than the mean for practice were considered as good, scores equal to the mean and less than the mean were considered as partial and bad practice, respectively.

Statistical analysis

Data were entered in an Excel sheet (Microsoft, Redmond, WA, USA) and SPSS version 24 (IBM Corp., Armonk, NY, USA) was used for analysis. Data analysis was done using percentages and ratio for descriptive data. The association between the variables was determined using the chi-square test. A p-value of < 0.05 was considered statistically significant.

## Results

Participant details

Questionnaires were shared with a total of 1830 students during the study period, out of which 902 students responded to the questionnaire with a response rate of 50.11%. Our study included 410 medical students, 337 nursing students and 155 allied health science students. The majority of the participants were female (n=739) and only 163 participants were male. The mean age of the participants was 20.23 years ±1.6 (minimum age - 17 years; maximum age - 29 years). Demographic details of the participants are given in Table 1.

**Table 1:** Demographic details of the participants MBBS: Bachelor of Medicine, Bachelor of Surgery; AHS: Allied Health Science

Age group (years)	MBBS		AHS		Nursing		
	Male	Female	Male	Female	Male	Female	
17-20	69	130	-	112	-		191
21-24	79	125	12	30	-		144
25-29	3	4		1	-		2

Knowledge

**Figure 1:**
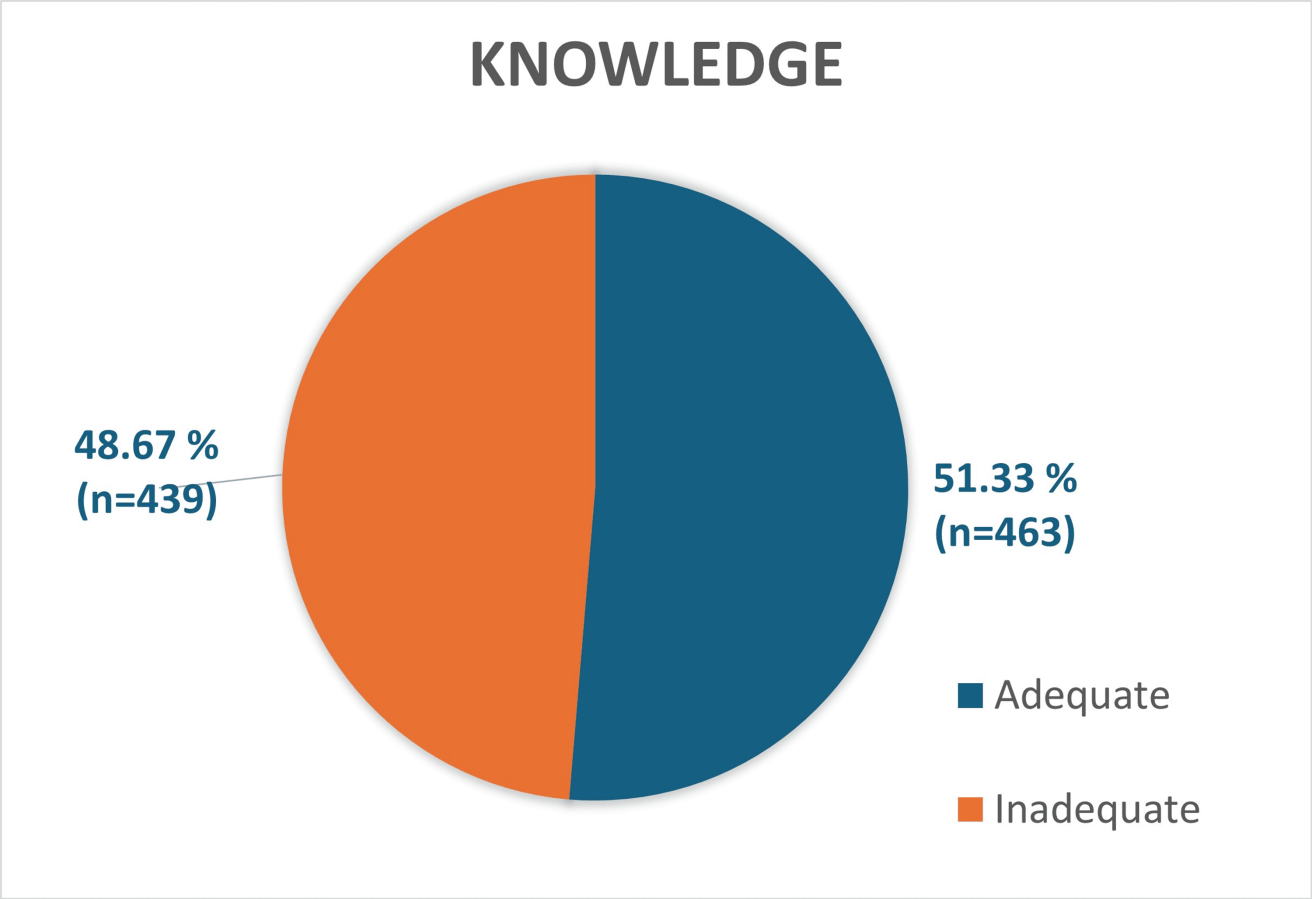
Distribution of knowledge level of students on gut microbiota

Mean score of knowledge for all participants assessed by MAS was 62±8.3 out of 92. Out of 902 participants in the study, more than half of the participants (51.33%; n=463) had adequate knowledge about the gut microbiota, whereas the remaining 439 (48.67%) participants had inadequate knowledge of gut microbiota as shown in Figure 1.

Maximum and minimum scores were 90 and 24, respectively. Among the total 163 male participants, 87 students (53.37%) had adequate knowledge and the remaining 76 students (46.62%) had inadequate knowledge. Among the female participants, 376 (50.87%) participants had adequate knowledge whereas the remaining 363 (49.12%) participants had inadequate knowledge on gut microbiota. There was no statistical significance between gender and knowledge, with a p value of 0.56 which was determined by chi square test. Among the three courses of health science students, more than half of the medical and allied health science students had adequate knowledge whereas among nursing students, only 147 students (43.62%) had adequate knowledge as depicted in Table 2.

**Table 2:** Distribution of knowledge on gut microbiota among the students of three courses MBBS: Bachelor of Medicine, Bachelor of Surgery

Course	Number of students with adequate knowledge n (%)	Number of students with Inadequate Knowledge n (%)
MBBS	230(56.09%)	180(43.90%)
Nursing	147(43.62%)	190(56.37%)
Allied Health Science	86(55.48%)	69(44.51%)

Course of study was statistically significant with knowledge, with a p value of 0.001646. MAS scores of first-year students were lower than remaining students as shown in Table 3.

**Table 3:** Percentage of students with adequate knowledge across years of study

Year of Study	Number of Students with adequate Knowledge n (%)
I	118(37.81)
II	96(56.6)
III	139(54.46)
IV	82(49.32)
Intern	28(54.71)

Attitude

**Figure 2:**
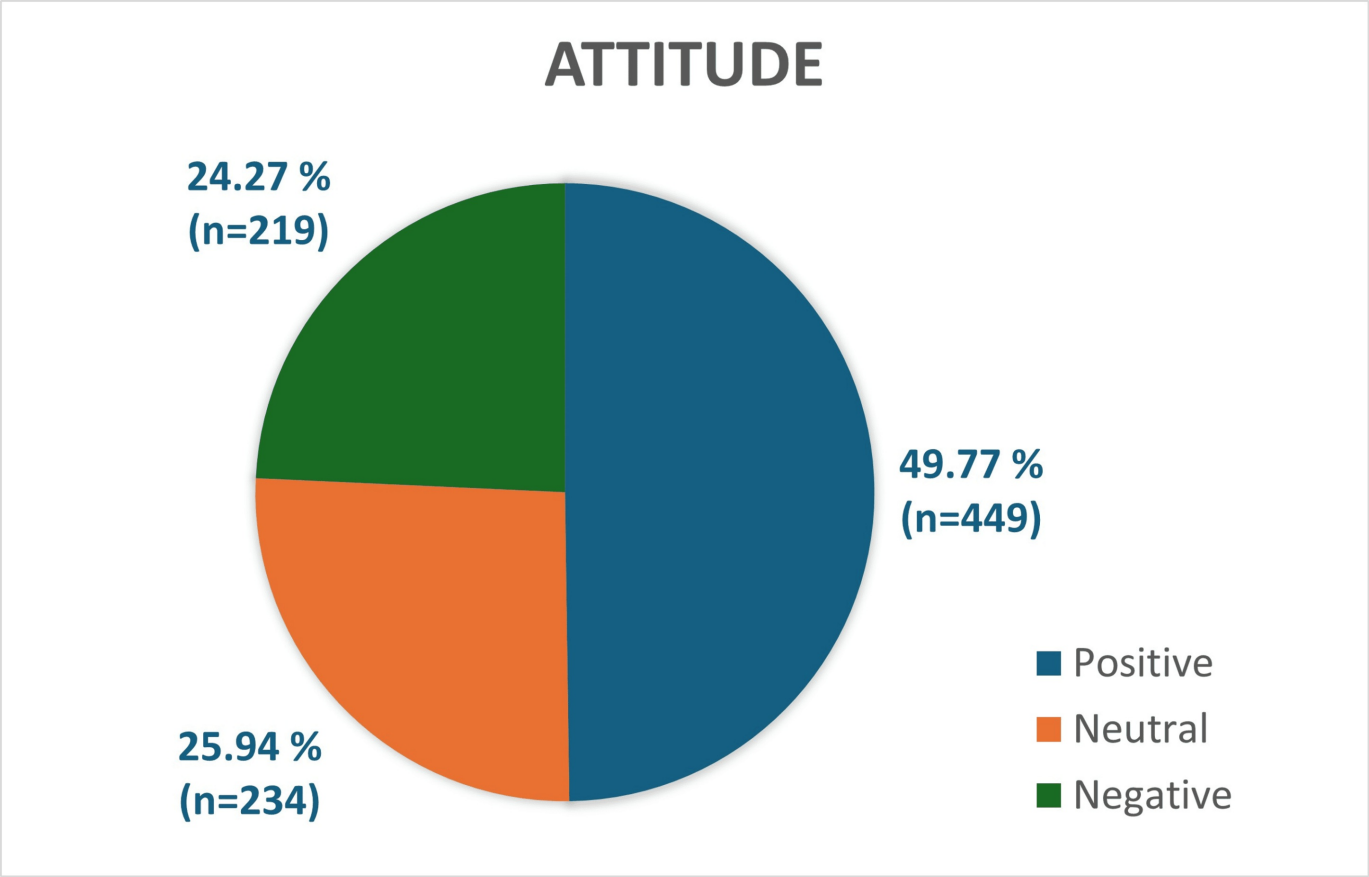
Distribution of attitude level of students on gut microbiota

Out of 902 participants, 449 students (49.77%) had positive attitude, 234 students (25.94%) had neutral and 219 students (24.27%) had negative attitude towards gut microbiota as shown in Figure 2.

Mean score of participants regarding attitude was 3.11±1.12. The maximum and minimum scores were 4 and 0, respectively. The distribution of attitude of students on gut microbiota across three courses is shown in Table 4. Course of study did not have significant difference in attitude, with a p value of 0.1233.

**Table 4:** Distribution of attitude on gut microbiota among the students of three courses MBBS: Bachelor of Medicine, Bachelor of Surgery; AHS: Allied Health Science

Course	Number of Students with Positive Attitude n (%)	Number of Students with Neutral attitude n (%)	Number of students with Negative Attitude n (%)
MBBS	224(54.63)	89(21.7)	97(23.6)
Nursing	141(41.83)	94(27.89)	102(30.2)
AHS	84(54.19)	51(32.9)	20(12.9)

Practices

**Figure 3:**
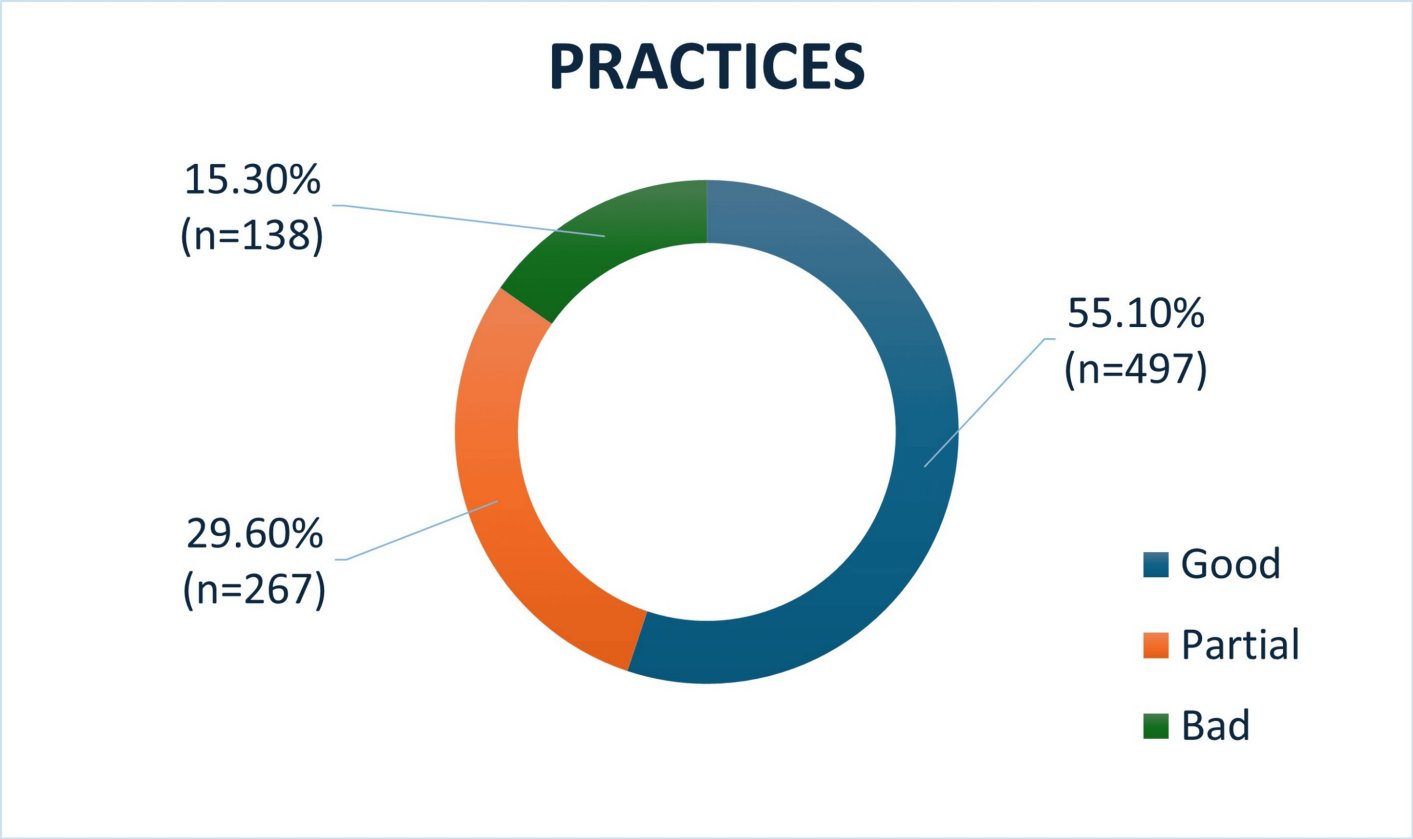
Distribution of practices of students on gut microbiota

Out of 902 participants, 497 students (55.09%) had good practice, 267 students (29.6%) had partial and 138 students (15.29%) had bad practices towards gut microbiota as shown in Figure 3.

Mean score of participants regarding attitude was 12.37±3.5. Maximum and minimum scores were 2 and 19, respectively. The distribution of practices of students on gut microbiota across three courses is shown in Table 5. Course of study did not have a significant difference in the practices, with a p value of 0.1072.

**Table 5:** Distribution of practices on gut microbiota among the students of three courses MBBS: Bachelor of Medicine, Bachelor of Surgery; AHS: Allied Health Science

Course	Number of students with Good Practices n(%)	Number of students with Partial practices n(%)	Number of students with Bad Practices n (%)
MBBS	266(64.8)	70(17.07)	74(18.04)
Nursing	145(43.02)	90(26.7)	102(30.26)
AHS	86(55.48)	35(22.5)	34(21.93)

## Discussion

Normal commensals are very important in maintaining the body's equilibrium. Normal commensals provide a high amount of benefits to the human body. Mainly, the gut microbiota plays an important role. So awareness of gut health and gut microbiota and their importance, along with their attitude and practice, was assessed in this study. A study conducted in Eastern Turkiye among university students showed that the MAS scores of females were higher compared to males [17]. Whereas a study done in Turkiye reported that knowledge about probiotics was higher among females than males [18]. The increase in female participants in the above two studies could be a cause for the significance. On the other hand, a study done in Jordan reported no significant difference in microbiota knowledge among male and female participants [19]. A study done among Trakya University students and a Turkey study among university students also did not find significant difference in MAS score among male and female participants [20,21]. In our study, despite more female participants, there was no significant difference in knowledge on gut microbiota between male and female students.

In medical education, nursing and allied health sciences, students study medical microbiology in the second professional year and are exposed to clinical postings from the second year of their course. A Serbian study [22] and studies from Turkiye found a significant difference in knowledge of gut microbiota among first-year and final-year students [17,23]. Fluctuations in MAS score across years of study were reported in the study among Trakya University students [20]. In our study, scores of first-year students were lower than the remaining students and fluctuations of the MAS score were noted among students across the years of study as shown in Table 3. This fluctuation in knowledge level on gut health and gut microbiota among students can be strengthened by implementing continuous training.

A study conducted by Abu et al. in Jordan found that health science students had better knowledge of microbiota when compared to other students pursuing non-health disciplines [19]. A study done in Serbia revealed that medical students had better knowledge of microbiota when compared to students pursuing pharmacy [22]. This finding correlated with our study where medical students had better knowledge compared to the other two courses. Both of these studies had their own self-structured questionnaire. There were very few studies that used MAS to assess the knowledge of students.

To the best of our search through the literature, there were very few studies that utilized the MAS score to assess microbiota awareness among health science students. The average score of students in our study was found to be 62±8.3 which coincides with the study done at Eastern Türkiye [17]. Whereas a study done by Davarci et al. had an average score of 70.3 [20] and this could be due to the fact that the above study was done only among medical students.

A Serbian study classified the participants, based on the number of questions answered correctly, into three groups and their results were 12.5% had good knowledge, 53.2% had fair knowledge and 34.2% had poor knowledge [22]. Whereas in a study done in Jordan, 39.1% of participants had good knowledge, 50.2% had basic knowledge and 10.7% had poor knowledge [19]. However, both of the above-mentioned studies did not use the MAS for knowledge measurement.

In our study, half of the participants had adequate knowledge when assessed with MAS scoring which correlated with the studies done by Davarci et al. [20] and Ozgenur Yilmaz et al. [24].

Attitude and practices

With respect to attitude and practices towards gut microbiota, the majority of the study population had a positive attitude (49.77%) and good practices (55.10%), which could contribute to maintaining gut health and microbiota like consuming probiotics and prebiotics regularly. These results coincide with the study done by Lakshmy et al. which included participants from Bangalore, Mumbai, Delhi and a study conducted by Das et al. in Delhi [25,26]. When it comes to the consumption of processed food, 34.4% of the participants of our study consume it on a regular basis. Health hazards caused by regular consumption of processed foods and the avoidance of regular consumption of processed foods should be addressed through proper health education to students.

A limitation of our study was that participants were from a single institute; recall bias and social desirability bias also could have played a role in their scores. As this study was conducted among health science students, results cannot be generalised.

## Conclusions

Half of the surveyed population had adequate understanding of gut microbiota and the remaining participants had inadequate knowledge on gut microbiota. Almost the same proportion of participants who had adequate knowledge also have positive attitudes and hence exhibit behaviors that support good gut health. This study emphasizes that bridging the knowledge gap would promote better attitudes and practices towards factors that influence gut microbiota and instigate a change in behavioral patterns towards gut health preservation. Educating and strengthening the knowledge would promote good attitude and practices of future healthcare workers and this would promote the same in the community. Studies on attitude and practices towards gut microbiota are very limited in the available literature and further research on these aspects are needed. Thorough understanding of the effects of dysbiosis on human health can be obtained by health education of the community, which may have a profound effect on the burden of non-communicable diseases in the future.

## Appendices

**Table 6:** Attitude and Practice Questionnaire

Attitude		Yes	No
	Are you willing to take probiotics?		
	Are you willing to take prebiotics?		
	Will you consume probiotics after an episode of diarrhea?		
	Will you consume probiotics for regularizing your bowel habits (in case of constipation)?		
Practices		Yes	No
1.	Do you consume probiotics regularly? If yes, how often? Once in a month Once in two weeks Once in a week Almost daily		
2.	Do you consume prebiotics regularly? If yes, how often? Once in a month Once in two weeks Once in a week Almost daily		
3.	Have you ever used probiotics during an episode of diarrhea?		
4.	Have you ever used probiotics for regularizing your bowel habits (in case of constipation)?		
5.	How do you take antibiotics and on what occasions? Bought directly from a pharmacy everytime there is an illness Bought from pharmacy only for certain illnesses sometimes Use a family member/friend’s prescription for a similar illness Visit a doctor and use the antibiotic whenever the illness arises Always visit a doctor and use currently prescribed antibiotics only		
6.	How often do you consume processed food? Everyday Almost daily Once in a week Once in two weeks Once in a month		

Scoring:

Attitude

If answered yes - 1 point, No - 0 points

Maximum score - 4 points

Minimum score - 0 points

Practice

Question 1&2 - No- 0 points; Yes- a) 1 point, b) 2 points, c) 3 points, d) 4 points

Question 3&4 - If answered Yes 1 point, No 0 points

Question 5 - a) 1 point, b) 2 points, c) 3 points, d) 4 points, e) 5 points

Question 6 - a) 1 point, b) 2 points, c) 3 points, d) 4 points, e) 5 points

Maximum score - 15 points

Minimum score - 2 points

Total Attitude + Practice

Maximum score - 4 + 15 = 19 points

Minimum score - 0 + 2 = 2 points
